# Jejunal atresia associated with idiopathic ileal perforation

**DOI:** 10.4103/0971-9261.43039

**Published:** 2008

**Authors:** P. C. Das, Rakesh Rai, Grover J. Lobo

**Affiliations:** Department of Pediatric Surgery, Fr. Muller Medical College and Hospital, Mangalore, Karnataka, India; 1 Department of General Surgery, Fr. Muller Medical College and Hospital, Mangalore, Karnataka, India

**Keywords:** Intestinal perforation, idiopathic ileal perforation, jejunoileal atresia, meconium peritonitis

## Abstract

Jejunoileal atresia is one of the common causes of neonatal intestinal obstruction. Intestinal perforation with meconium peritonitis in the neonatal period, which carries a high mortality rate, is also common. The association of jejunal atresia with idiopathic ileal perforation is very rare.

## INTRODUCTION

Bowel obstruction in the newborn is one of the most common surgical problems, and its successful management depends upon both timely diagnosis and prompt therapy. Intestinal atresia is one of the three common causes of neonatal intestinal obstruction, the other two being Hirschsprung's disease and anorectal malformation. Among the types of intestinal atresias, jejunoileal atresia is the most common one. Unlike duodenal atresia, it is less commonly associated with Down's syndrome, malrotation and congenital cardiac defects.[1] We report a case of jejunal atresia associated with distal ileal perforation. On search of English literature, we find only one case of atresia with perforation in distal atretic segment.[2]

## CASE REPORT

**Figure 1:**
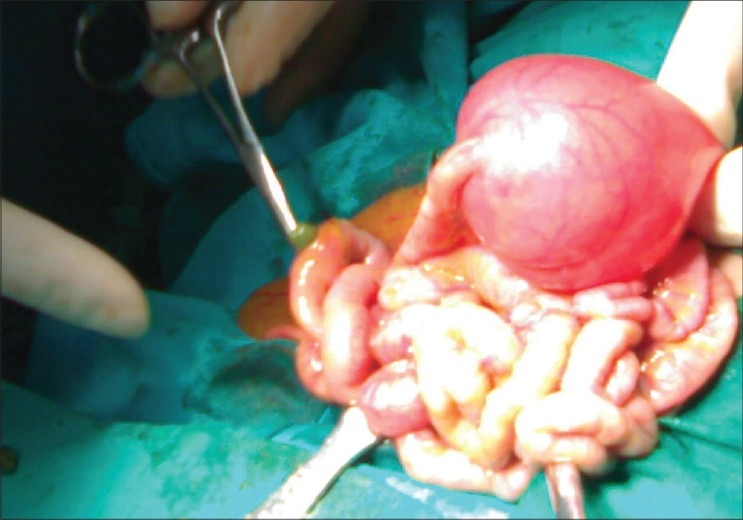
Proximal jejunal atresia (type-1). The tip of the artery forceps indicates the site of distal ileal perforation

A full-term male neonate weighing 2.8 kg was born by spontaneous vaginal delivery. The child cried immediately after birth. Antenatal sonogram showed dilated bowel loops suggestive of intestinal obstruction. The mother did not had polyhydramnios during pregnancy. The child was admitted to the neonatal intensive care unit. He was active, pink and stable. Cardiovascular system was normal. He had upper abdominal distension without any mass. An abdominal erect x-ray showed three air-fluid levels in the upper half of abdomen with the distal half being gasless. No free air was found in the abdomen. Renal function test was normal. With the diagnosis of jejunal atresia, the patient underwent laparotomy on the second day. During laparotomy, type-1 jejunal atresia was found at approximately 10 cm distal to the D-J flexure, and a single distal ileal perforation was observed on the antimesenteric side with evidence of meconium peritonitis [Figure 1]. The entire part of the gut distal to the atresia was unused and collapsed. Further, there was no evidence of gut inflammation. Jejunal atresia was treated by tapering jejunoplasty and end-to-side jejuno-jejunostomy by using a transgastric jejunostomy infant feeding tube. Ileal perforation was treated with resection and anastomosis. Feeding was started through the transgastric jejunostomy tube on the 4th post-operative day. Postoperative recovery was uneventful. The baby was discharged after two weeks. Histopathological examination of the resected perforated ileum showed single perforation with congestion and hemorrhage in the submucosa. There was no evidence of necrotizing enterocolitis (NEC). Ganglion cells were present in the resected segments.

## DISCUSSION

Jejunoileal atresia is the most common type of intestinal atresia. There are several reports of non-NEC-related neonatal intestinal perforation in the literature.[3–5] Sajja *et al.*[2] reported the first case of duodenal atresia associated with idiopathic small bowel perforations.[2] Possibly, our case is the second a similar case report (perforation distal to the site of atresia). It is not yet proved whether idiopathic (non-NEC-related) bowel perforation with intestinal atresia proximal to the site of perforation share a common etiology or it is just a coincidence. Jejunoileal atresia is caused by mesenteric vascular accident during the fetal life, whereas duodenal atresia is due to the failure of vacuolization from the solid-cord stage of development.[1,6,7] The etiology of idiopathic intestinal perforation is not completely known. Various causes suggested for the idiopathic intestinal perforation are the absence or thinning of muscularis propria, absence of muscular layer, excessive resorption of muscularis as a part of regression of the omphalomesenteric duct and localized ischemic process.[2,8] Intrauterine cocaine exposure has been reported as a cause of non-NEC-related intestinal perforation.[9] The site of perforation of idiopathic intestinal perforation has been reportedly found in the stomach, jejunum, ileum and colon – ileum being the most common site.[2] Diagnosis of intestinal perforation with proximal intestinal atresia is only possible during laparotomy. The classical finding of pneumoperitoneum will never be observed in these types of cases by using the following techniques: x-ray ultrasound, and CT abdomen. This is because of the presence of proximal atresia. Mortality and morbidity of idiopathic intestinal perforation with meconium peritonitis remains high.[10,11] Prognosis of patients with intestinal atresia with distal idiopathic intestinal perforation will be worse if it is not properly treated during preoperative, operative and postoperative period. Today, mortality and morbidity of these cases can definitely be reduced with good ICU care, advanced anesthesia, proper antibiotics, early surgery and total parenteral/enteral nutrition.
